# Determinant Factors of Poor Hygiene Behaviors in Mongolian Adolescents

**DOI:** 10.7759/cureus.105791

**Published:** 2026-03-24

**Authors:** Javzan Badarch, Nyamdelger Bat-Orshikh, Tserenkhand Zorigtbaatar, Bolormaa Sainbayar, Bayar Chuluunbaatar

**Affiliations:** 1 Department of Dental Hygiene, Mongolian National University of Medical Sciences, Ulaanbaatar, MNG; 2 Department of Dental Hygiene, School of Dentistry, Mongolian National University of Medical Sciences, Ulaanbaatar, MNG; 3 Department of Product Research, Monitoring, and Inspection, School of Animal Science and Biotechnology, Mongolian University of Life Sciences, Ulaanbaatar, MNG; 4 Department of Orthodontics, School of Dentistry, Mongolian National University of Medical Sciences, Ulaanbaatar, MNG; 5 Department of Drug Registration, Medicine and Medical Devices Regulatory Authority, Ulaanbaatar, MNG

**Keywords:** adolescents, hand hygiene, mongolia, oral hygiene, prevalence

## Abstract

Introduction

Good hygiene behaviors in school develop lifetime positive behaviors in communities. The purpose of the study was to demonstrate poor oral and hand hygiene behaviors and their associated factors among adolescents in Mongolia.

Methods

The survey involved 7149 Mongolian school-aged adolescents who took part in the “Global School-based Student Health Survey” in 2019.

Results

Altogether, 35.6% (N = 2542) of students reported poor oral hygiene. Suboptimal hand hygiene practices were common among adolescents: 69.2% (N = 1754) before meals, 72.2% (N = 1827) after toilet use, and 47.0% (N = 1187) reported not always using soap. In the multivariate logistic regression analysis, being male, students who live in rural areas, inadequate dietary behaviors including fruit and vegetable consumption, health risk behaviors such as parental smoking, exposure to secondhand smoke, physical inactivity, and sedentary leisure time were associated with poor oral and hand hygiene.

Conclusion

Significant proportions of insufficient tooth brushing and hand-washing practices were found among school-attending children in Mongolia. Numerous risk factors (demographics, dietary behaviors, and health risk behaviors) for poor oral and hand hygiene were identified, which can be utilized for intervention programs for the youth population.

## Introduction

Maintaining proper oral and hand hygiene is critical to preventing infectious diseases such as dental caries, diarrhea, and respiratory diseases and promoting overall health, especially among young populations [1]. Maintaining proper hygiene is essential for general health and well-being, especially in young people. Healthy practices formed during these early stages often persist into adulthood [2]. The importance of having adequate sanitation facilities is that children who learn in a clean and hygienic environment grow up healthy, which contributes to academic success and reduces dropout rates [3].

Oral health is still one of the major public health issues globally. The World Health Organization (WHO) estimates that globally 3.5 billion people suffer from dental and oral diseases, with a significant proportion of cases occurring in South-East Asian and Western Pacific region countries [4]. Brushing teeth at least twice a day is strongly recommended by dental care providers, as it plays a crucial role in preventing dental plaque and oral diseases [5]. According to a survey performed by the Japan International Cooperation Agency (JICA) partnership program in Mongolia, 81% of the children under five years of age suffer from dental caries [6]. The National Oral Health Program conducted in Mongolia from 2006 to 2015 reported that the prevalence of dental caries among children under six years of age was 83.9%, with 90-92% requiring dental treatment [7], which shows us that dental caries is one of the most prevalent diseases among Mongolian children.

The WHO highlights that maintaining proper hand hygiene through regular and thorough cleaning with soap and water is an essential preventive practice to control infectious disease spread [8]. In 2021, 58% of schools had a handwashing facility with soap and water available, 17% had a handwashing facility with water but no soap available, and 25% had no facilities or no water at the school [9]. A study utilizing data from the Global School-based Student Health Survey (GSHS) between 2003 and 2017 found that approximately two-thirds of adolescents engaged in inadequate hand hygiene practices [10]. Enkhbat et al. demonstrated that only 50.1% of 5th-grade students washed their hands before eating and after using the toilet, and only 34% used soap while at school in 2019 [11]. In Mongolia, “Norms and Requirements for WASH in Schools, Dormitories, and Kindergartens” was approved in June 2015 by the Minister of Education, Culture, and Science, the Minister of Health and Sport, and the Minister of Finance as a result of a series of advocacy activities by the United Nations Children's Fund (UNICEF) in cooperation with partners [12].

Several international studies have shown that numerous determinants are linked with poor oral and hand hygiene practices among adolescents, such as male gender [13], older grade student [14], rural location [15], inadequate fruit/vegetable intake [16], health risk behaviors such as cigarette smoking [17], parental smoking [18], and inadequate exercise/leisure time sedentary behavior [19]. There is insufficient recent information on hygiene behaviors, including oral and hand hygiene, their prevalence, and association with dietary and health risk behaviors among Mongolian school-aged adolescents. Therefore, the aim of the current study is to examine and assess the prevalence and correlates of poor oral and hand hygiene practices among adolescents in Mongolia.

## Materials and methods

Sampling procedure

The GSHS used a two-stage cluster sampling design where participants were given a self-reported questionnaire. In the first stage, the probability of schools being selected was proportional to the number of students enrolled. The second stage involved classes being randomly selected and all students in the selected classes being eligible to participate [20]. Altogether, 7149 students participated in the 2019 Mongolian GSHS survey. Students were asked to participate voluntarily in the survey, and written informed consent was obtained from each student and their parents/guardians. Participants were selected from the Mongolian dataset of the GSHS-2019. Students aged 10-18 years who completed the survey and had available data from the variables analyzed in this study were included. Respondents with missing, incomplete, or inconsistent responses on the variables of interest were excluded from the analysis. This study procedure was approved by the Research Ethical Committee of Mongolian National University of Medical Sciences (2024/3-02).

Data source

The dataset used in the present study was previously utilized in a peer-reviewed publication by Badarch et al. [21], which examined suicide attempts and associated factors among school-attending adolescents in Mongolia.

Measures

Oral and hand hygiene behaviors were assessed with four questions. With oral hygiene, students were asked, “During the past 30 days, how many times per day did you usually clean or brush your teeth?" Response options were as follows: 1 = I did not clean or brush my teeth during the past 30 days; 2 = less than one time per day; 6 = four or more times a day. A dichotomous variable was created where one to three responses were coded as “0” and four to six responses were coded as “1.” The codes 0 and 1 represented poor oral hygiene and good oral hygiene. Hand hygiene was derived from three questions: (1) “During the past 30 days, how often did you wash your hands before eating?”; (2) “During the past 30 days, how often did you use soap when washing your hands?”; and (3) “During the past 30 days, how often did you wash your hands after using the toilet or latrine?” Response options included the following: 1 = never, 2 = rarely, 3 = sometimes, 4 = most of the time, and 5 = always. Each question was dichotomously recoded as never/rarely/sometimes/most of the time = “no” and always = "yes." The estimations contained 10 explanatory variables. Gender, grade, location, fruit and vegetable consumption, current cigarette smoking, parental smoking, exposure to second-hand smoke (SHS), physical activity, and sedentary behavior were among the factors considered. With the exception of grade, all variables were dichotomized as yes or no/good or poor answers. Detailed descriptions of the variables are presented in Table 1.

**Table 1:** Description of the study variables.

	Variables	Question	Response options	Coding scheme
Hygiene behaviors	Oral hygiene	“During the past 30 days, how many times per day did you usually clean or brush your teeth?”	‘1’ = I did not clean or brush my teeth during the past 30 days, to ‘6’ = 4 or more times per day	1-3 = poor and 4-6 = good)
	Hand washing (before meals)	“During the past 30 days, how often did you wash your hands before eating?”	‘1’ = never to ‘5’ = always	1-4 = poor and 5 = good
	Hand washing (after toilet)	“During the past 30 days, how often did you wash your hands after using the toilet or latrine?”	‘1’ = never to ‘5’ = always	1-4 = poor and 5 = good
	Hand washing (with soap)	“During the past 30 days, how often did you use soap when washing your hands?”	‘1’ = never to ‘5’ = always	1-4 = poor and 5 = good
Demographics	Gender	“What is your sex?”	‘1’ = male, and ‘2’ = female	0 = male and 1 = female
	Grade	“In what grade are you?”	‘1’ = 5^th^ grade to ‘8’ = 12^th^ grade	1 = elementary, 2-5 = middle, and 6-8 = high
	Location	“Where do you live?"	‘1’ = urban and ‘2’ = rural	0 = urban and 1 = rural
Dietary behaviors	Fruits intake	“During the past 30 days, how many times per day did you usually eat fruit such as apples, bananas, and oranges?”	‘1’ = I did not eat fruit during the past 30 days to ‘7’ = 5 or more times per day	1-3 = inadequate and 4-7 = adequate
	Vegetables intake	“During the past 30 days, how many times per day did you usually eat vegetables, such as salads, spinach, eggplant, tomatoes, and cucumbers?”	‘1’ = I did not eat vegetables during the past 30 days to ‘7’ = 5 or more times per day	1-4 = inadequate and 5-7 = adequate
Health risk behaviors	Current cigarette smoking	“During the past 30 days, on how many days did you smoke cigarettes?”	‘1’ = 0 day to ‘7 ’= all 30 days	2-7 = yes and 1 = no
	Parental smoking	“Which of your parents or guardians uses any form of tobacco?”	‘1’ = neither to ‘4’ = both	2-4 = one or both and 1 = none
	Exposed to second-hand smoke	“During the past 7 days, on how many days did someone smoke in your presence?”	‘1’ = 0 day to ‘5’ = 7 days	2-5 = yes and 1 = no
	Physical activity	“During the past 7 days, on how many days were you physically active for a total of at least 60 minutes per day?”	‘1’ = 0 day to ‘8’ = 7 days	1 = inactive and 2-8 = active
	Sedentary behavior	“How much time do you spend during a typical or usual day sitting and watching television, playing computer games, talking with friends, or doing other sitting activities?”	‘1’ = less than 1 hour per day to ‘8’ = more than 8 hours per day	3-6 = yes and 1-2 = no

Data analysis

Data analysis was performed using IBM SPSS for Windows, version 28.0 (IBM Corp., Armonk, NY). Descriptive statistics were done to describe the study sample; percent distributions were calculated on the number of respondents. Factors associated with poor oral and hand hygiene were examined at both the univariate and multivariable levels using univariate and multivariable logistic regression analyses, respectively. Univariate logistic regression analysis was conducted to examine unadjusted associations between oral and hand hygiene and independent variables. Multivariable logistic regression analysis was used to assess the independent contribution of demographics, dietary behaviors, and health risk behaviors to poor oral and hand hygiene practices. The independent variables involved in the regression analysis were sex, student grade, location, inadequate fruit and vegetable intake, cigarette smoking, parental smoking, exposure to SHS, being physically inactive, and sitting more than three hours a day. Odds ratio (OR) and 95% CI of OR were used to indicate the association between poor oral and hand hygiene and the designated independent variables. Statistical significance was defined at p < 0.05.

## Results

Sample characteristics and hygiene behaviors

Table 2 presents the sample characteristics of 7149 participants who took part in Mongolian GSHS 2019. Middle school students (between 6 and 9 grades) were 46% (N = 3274). Male and female participants were relatively balanced, with 45.8% (N = 3258) boys and 54.2% (3861) girls (30 respondents were missed for this question). The majority of the participants were from rural areas (74.6%, N = 5334), compared to 25.4% (N = 1815) from urban areas. Of the respondents with poor oral hygiene, 69.2% (N = 1754) did not wash their hands before eating, 72.2% (N = 1827) did not wash their hands after using the toilet, and 47% (N = 1187) did not use soap to wash their hands. The characteristics of the sample are shown in Table 2.

**Table 2:** Sample characteristics and oral and hand hygiene behaviors.

Characteristics		Poor oral hygiene	Poor hand hygiene		
			Before meals	After toilet	With soap
		Number (%)	Number (%)	Number (%)	Number (%)
Hygiene behaviors	Oral hygiene				
	Poor	-	1754 (69.2)	1827 (72.2)	1187 (47.0)
	Good	-	2445 (53.3)	2564 (55.9)	1628 (35.5)
	Hand washing before eating				
	No	1754 (41.8)	-	3193 (76.2)	1992 (47.6)
	Yes	779 (26.6)	-	1194 (41.0)	813 (27.9)
	Hand washing after using the toilet				
	No	1827 (41.6)	3193 (72.8)	-	2025 (46.2)
	Yes	702 (25.8)	1000 (36.8)	-	787 (29.0)
	Hand washing with soap				
	No	1187 (42.2)	1992 (71.0)	2025 (72.0)	-
	Yes	1341 (31.2)	2196 (51.1)	2356 (55.0)	-
Demographics	Gender				
	Male	1405 (43.2)	2014 (62.0)	2150 (66.4)	1354 (41.8)
	Female	1125 (29.2)	2174 (56.4)	2231 (57.9)	1453 (37.8)
	Grade				
	Elementary school	474 (32.5)	675 (46.4)	764 (52.7)	628 (43.3)
	Middle school	1199 (36.7)	1912 (58.6)	2070 (63.4)	1294 (39.7)
	High school	857 (35.9)	1601 (67.2)	1547 (65.1)	883 (37.1)
	Area				
	Urban	645 (35.6)	1084 (60.0)	1027 (56.8)	689 (38.2)
	Rural	1897 (35.6)	3119 (58.6)	3371 (63.5)	2129 (40.0)
Dietary behaviors	Fruit intake				
	Inadequate	2198 (37.0)	3660 (61.7)	3768 (63.6)	2349 (39.7)
	Adequate	337 (28.3)	533 (45.0)	617 (52.2)	461 (38.9)
	Vegetable intake				
	Inadequate	1305 (39.0)	2124 (63.6)	2231 (67.0)	1437 (43.2)
	Adequate	1229 (32.6)	2061 (54.8)	2151 (57.1)	1370 (36.4)
Health risk behaviors	Current cigarette smoking				
	Yes	556 (39.7)	871 (62.2)	865 (61.8)	590 (42.2)
	No	1972 (34.5)	3318(58.2)	3514 (61.7)	2214 (38.9)
	Parental smoking				
	One or both	1108 (36.9)	1870 (62.2)	1897 (63.3)	1198 (39.9)
	None	1227 (33.2)	2044 (55.4)	2196 (59.6)	1410 (38.2)
	Exposed to second-hand smoke				
	Yes	1597 (37.7)	2609 (61.6)	2637 (62.3)	1727 (40.8)
	No	923 (32.4)	1564 (55.0)	1730 (60.9)	1070 (37.7)
	Physical activity				
	Inactive	557 (42.4)	787 (59.9)	872 (66.6)	596 (45.5)
	Active	1969 (34.0)	3389 (58.7)	3498 (60.6)	2207 (38.2)
	Sedentary behavior				
	Yes	1056 (37.6)	1817 (64.8)	1793 (639)	1062 (37.9)
	No	1457 (34.2)	2330 (54.8)	2549 (60.1)	1720 (40.5)

**Figure 1:**
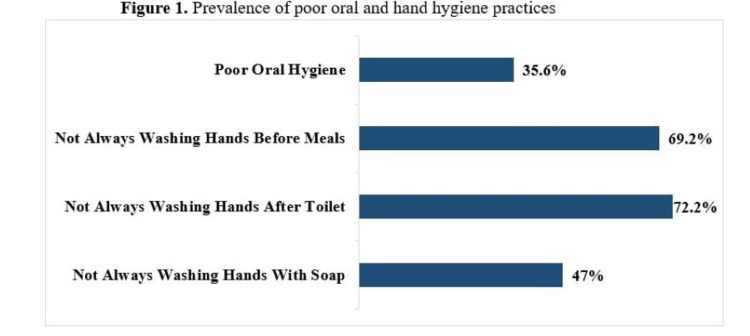
Prevalence of poor oral and hand hygiene practices.

Figure 1 presents the frequency of poor hygiene practices. The prevalence of insufficient tooth brushing was 35.6% (N = 2542) among school-attending adolescents. Not always washing hands before meals, not always washing hands after toilet, and not always washing hands with soap were 69.2% (N = 1754), 72.2% (N = 1827), and 47% (N = 1187), respectively.

In accordance with univariate analysis (Table 3), students who brush their teeth less than twice a day were more likely to be males, older grade students, have inadequate fruit and vegetable intake, be current smokers, have parental smoking, be exposed to SHS, be physically inactive, and engage in sedentary behavior. Adolescents who have poor hand hygiene practices, including before meals, after the restroom, and with soap, were more likely to be males, older grade students, have insufficient fruit and vegetable consumption, be current smokers, have parental smoking, be exposed to SHS, physical inactivity, and leisure time sedentary behavior. The highest odds were found in connection with the male gender, especially older grade students, inadequate vegetable intake, and sedentary behavior.

**Table 3:** Univariate logistic regression of poor oral and hand hygiene behaviors. Notes: *** p < 0.001; ** p < 0.01; * p < 0.05. UAOR: unadjusted odds ratio; 95% CI: 95% confidence interval.

Characteristics	Poor oral hygiene, UAOR (95% CI)	Poor hand hygiene		
		Before meals, UAOR (95% CI)	After toilet, UAOR (95% CI)	With soap, UAOR (95% CI)
Demographics				
Gender/male	1.8 (1.67-2.04)***	1.2 (1.14-1.38)***	1.4 (1.30-1.58)***	1.1 (1.07-1.30)***
Increase 1 grade	1.1 (1.01-1.33)*	2.3 (2.07-2.70)***	1.6 (1.47-1.91)***	1.1 (1.0-1.24)*
Area/rural	1.0 (0.89-1.11)	1.0 (0.95-1.18)	1.3 (1.18-1.47)***	0.9 (0.82-1.03)
Dietary behaviors				
Inadequate fruit intake	1.4 (1.29-1.70)***	1.9 (1.73-2.23)***	1.5 (1.41-1.81)***	1.0 (0.90-1.17)
Inadequate vegetable intake	1.3 (1.20-1.46)***	1.4 (1.31-1.58)***	1.5 (1.38-1.67)***	1.3 (1.20-1.45)***
Health risk behaviors				
Current cigarette smoking	1.2 (1.10-1.40)***	1.1 (1.05-1.33)**	1.0 (0.89-1.13)	1.1 (1.02-1.29)**
One or both parents smoke	1.1 (1.06-1.30)**	1.3 (1.20-1.46)***	1.1 (1.05-1.29)**	1.0 (0.97-1.18)
Exposed to SHS	1.2 (1.14-1.39)***	1.3 (1.19-1.45)***	1.0 (0.96-1.17)	1.1 (1.03-1.25)**
Physically inactive	1.4 (1.26-1.61)***	1.0 (0.93-1.19)	1.2 (1.14-1.46)***	1.3 (1.19-1.52)***
Sedentary behavior	1.1 (1.04-1.27)***	1.5 (1.37-1.67)***	1.1 (1.06-1.29)**	1.1 (1.01-1.23)**

Table 4 shows multivariate logistic regression models of factors associated with poor oral and hand hygiene behaviors in Mongolian adolescents. Male students were 1.8 times (adjusted odds ratio (AOR) = 1.87; 95% CI: 1.67-2.04) more likely to have poor oral hygiene than females. Moreover, inadequate dietary behaviors, including fruit and vegetable intake, were 1.4 times (AOR = 1.42; 95% CI: 1.29-1.70) and 1.3 times (AOR = 1.31; 95% CI: 1.20-1.46) as likely as to have insufficient tooth brushing. Students whose parents were smokers were 11% (AOR = 1.11; 95% CI: 1.14-1.39), and students who were exposed to SHS were 13% (AOR = 1.13; 95% CI: 1.14-1.39) more likely to report suboptimal tooth brushing. Physically inactive students were 1.4 times (AOR = 1.40; 95% CI: 1.26-1.61) and students who spent more than three hours sitting were 1.1 times (AOR = 1.19; 95% CI: 1.04-1.27) as likely as their counterparts to practice poor oral hygiene.

**Table 4:** Multivariate logistic regression of poor oral and hand hygiene behaviors. *** p < 0.001; ** p < 0.01; * p < 0.05. AOR: adjusted odds ratio.

Characteristics	Poor oral hygiene, AOR (95% CI)	Poor hand hygiene		
		Before meals, AOR (95% CI)	After toilet, AOR (95% CI)	With soap, AOR (95% CI)
Demographics				
Gender/male	1.87 (1.68-2.07)***	1.29 (1.17-1.43)***	1.46 (1.32-1.62)***	1.15 (1.04-1.27)**
Increase one grade				
Area/rural	0.91 (0.80-1.04)	1.05 (0.93-1.19)	1.31 (1.15-1.48)***	0.90 (0.79-1.02)
Dietary behaviors				
Inadequate fruit intake	1.42 (1.22-1.64)***	1.78 (1.55-2.03)***	1.45 (1.27-1.66)***	1.01 (0.88-1.16)
Inadequate vegetable intake	1.26 (1.13-1.40)***	1.37 (1.24-1.52)***	1.35 (1.22-1.50)***	1.30 (1.17-1.44)***
Health risk behaviors				
Current cigarette smoking	1.10 (0.95-1.28)	1.03 (0.88-1.19)	1.05 (0.91-1.22)	1.10 (0.95-1.27)
One or both parents smoke	1.11 (1.00-1.23)*	1.20 (1.08-1.34)***	1.14 (1.02-1.26)*	1.06 (0.95-1.17)
Exposed to second-hand smoke	1.13 (1.00-1.27)*	1.15 (1.02-1.29)*	1.00 (0.89-1.24)	1.12 (1.00-1.26)*
Physically inactive	1.40 (1.22-1.59)***	1.10 (0.96-1.25)	1.27 (1.11-1.46)***	1.33 (1.17-1.51)***
Sedentary behavior	1.19 (1.07-1.33)**	1.42 (1.28-1.58)***	1.20 (1.08-1.33)**	1.11 (1.00-1.23)*

Boys were 1.2 times (AOR = 1.29; 95% CI: 1.14-1.38) more likely not to always have their hands washed before meals. Inadequate fruit and vegetable consumption was 1.9 times (AOR = 1.98; 95% CI: 1.73-2.23) and 1.4 times (AOR = 1.47; 95% CI: 1.31-1.58) as likely as to report not always washing hands before meals compared to their counterparts. In addition, students whose parents where smokers were 30% (AOR = 1.30; 95% CI: 1.20-1.46), students exposed to SHS were 35% (AOR = 1.35; 95% CI: 1.19-1.45), and students spent sitting more than three hours were 52% (AOR = 1.22; 95% CI: 1.37-1.67) more likely to report suboptimal hand practices such as before meals.

Male students were 1.4 times (AOR = 1.46; 95% CI: 1.30-1.58), and students who lived in rural areas were 1.3 times (AOR = 1.31; 95% CI: 1.18-1.47) as likely as to report not washing hands after using the restroom. Inadequate fruit and vegetable consumption was 55% (AOR = 1.55; 95% CI: 1.41-1.81), and 52% (AOR = 1.52; 95% CI: 1.38-1.67) were more likely to report not always washing hands after latrine. Meanwhile, students whose parents were smokers were 1.1 times (AOR = 1.14; 95% CI: 1.05-1.29), physically inactive students were 1.1 times (AOR = 1.27; 95% CI: 1.14-1.46), and students who spent sitting more than three hours were 1.1 times (AOR = 1.10; 95% CI: 1.06-1.29) as likely as to report not always washing hands after toilet compared to the counterparts.

In terms of not always washing hands with soap, in multivariate analysis, gender (male: AOR = 1.15; 95% CI: 1.04-1.27), dietary behavior (inadequate fruit consumption: AOR = 1.30; 95% CI: 1.17-1.44), health risk behaviors (physically inactive: AOR = 1.33; 95% CI: 1.17-1.51), and sedentary behavior (AOR = 1.11; 95% CI: 1.00-1.23) increased the risk of sub-optimal washing hands with soap.

## Discussion

Using data from the Mongolian GSHS-2019, we examined poor hygiene behaviors, including oral and hand hygiene, and their associated factors among adolescents in Mongolia. The prevalence of 35.6% of infrequent tooth brushing seemed higher than in previous studies (2016-2017) in three Caribbean countries (16.9%) [16] and in Malaysia (12.7%) [22]. Nevertheless, this result was lower than in Nepal (51.7%) and in Bhutan (58.3%) in 2015-2016 [23]. Compared with the results of the Mongolian GSHS-2013, the prevalence of poor oral hygiene (33%) [24] was slightly increased to 35.6% in this Mongolian GSHS-2019.

The prevalence of poor hand hygiene (before meals) (62.9%) was higher than in Lebanon (31.1%) [25], and Indonesia (46.9%), but lower than in Thailand (65.9%) [26], and China (78.06%) [25]. Poor hand hygiene (after toilet) (72.9%) was higher than in Ghana (36.4%) [27] and in Myanmar (20.4%) [17]. Poor hand hygiene (with soap) (47%) was lower than in Morocco (55.9%) [28] and in Afghanistan (51.8%) [29].

Consistent with previous research, this study revealed that suboptimal tooth brushing was more prevalent among males than females. Various factors may contribute to this inequality. Gender-based behavioral differences may also influence the frequency of tooth brushing, with girls always demonstrating greater concern for appearance and hygienic habits [30]. These findings highlight the importance of implementing oral health promotion approaches tailored to gender differences in school-based interventions.

Our results align with previous research, where inadequate fruit and vegetable consumption was associated with less frequent tooth brushing and handwashing. Unhealthy dietary behaviors and poor hygiene practices, including tooth brushing and hand hygiene, often coexist in the same individuals [31]. Dietary behaviors and personal hygiene practices established during a young age are critical determinants of long-term health outcomes.

The present study aligns with previous research indicating that parental smoking and SHS exposure were significantly associated with suboptimal oral and hand hygiene behaviors among adolescents. A nationally representative study in Mongolia reported in 2013 that school-attending adolescents exposed to parental smoking or SHS were less likely to have poor oral hygiene compared to their unexposed peers [24]. Smoking parents were less likely to provide the supervision necessary for establishing healthy routines in children [32].

We found that adolescents who reported a physically inactive and sedentary lifestyle were significantly more likely to exhibit poor hygiene practices, including infrequent tooth brushing and handwashing. This association may be attributed to overall health-compromising behaviors commonly observed among sedentary young people, such as reduced health awareness or lower engagement in personal hygiene routines. These results are consistent with previous studies utilizing data from the GSHS, which similarly reported a correlation between physically inactive/sedentary behavior and suboptimal hygiene behaviors [22,33]. Such results underscore the importance of integrated health promotion strategies that address both physical activity and hygiene education among school-aged children.

Many countries have implemented comprehensive school-based health promotion programs aimed at enhancing both oral and general health among children. These initiatives often include structured policies related to nutrition, tobacco prevention, and physical activity, and are designed to be accessible across both urban and rural settings. By fostering supportive environments that encourage healthy eating, regular physical activity, and awareness of harmful health behaviors, such programs play a critical role in establishing positive hygiene practices, including oral and hand hygiene from an early age.

## Conclusions

The findings indicated that tooth brushing and hand hygiene practices remain inadequate among Mongolian adolescents. School-aged adolescents who were males, lived in rural locations, had inadequate dietary behaviors, had health risk behaviors including parental smoking, were exposed to SHS, and had physically inactive and sedentary behavior were more likely to have poor oral and hand hygiene practices among Mongolian adolescents. Understanding the root cause of inadequate tooth brushing and suboptimal hand hygiene practices is crucial for designing targeted interventions and educational strategies to encourage young people to adopt healthier oral and general healthcare practices. Hygiene practices of school-attending adolescents may be improved through school-based programs that demonstrate proper hygiene techniques and schedule time during the school day for hygiene practices.
